# Hierarchical Contrastive Learning for Protein–Protein Interaction Prediction Across Organisms

**DOI:** 10.3390/ijms27146242

**Published:** 2026-07-13

**Authors:** Shiyi Liu, Buwen Liang, Yuetong Fang, Zixuan Jiang, Renjing Xu

**Affiliations:** 1 Function Hub, The Hong Kong University of Science and Technology (Guangzhou), Guangzhou 511453, China; sliudd@connect.ust.hk (S.L.); yfang870@connect.hkust-gz.edu.cn (Y.F.); zixuanjiang@hkust-gz.edu.cn (Z.J.); 2 The Hong Kong University of Science and Technology, Clear Water Bay, Kowloon, Hong Kong, China; 3 College of Information and Electrical Engineering, China Agricultural University, Beijing 100083, China; lbw124765283@126.com

**Keywords:** protein–protein interaction, contrastive learning, cross-species prediction, protein family, host–pathogen interaction

## Abstract

With advances in biomedical technologies and the continued expansion of experimental resources, biological data are growing rapidly in both scale and complexity. Contrastive learning provides an effective framework for integrating heterogeneous biological information. However, many protein–protein interaction (PPI) prediction methods still represent protein sequences and annotations as flat features and do not explicitly model hierarchical biological relationships among protein families, clans, and functional annotations. Here, we introduce **HIPPO** (**HI**erarchical **P**rotein–**P**rotein interaction prediction across **O**rganisms), a hierarchical contrastive learning framework for PPI prediction. HIPPO aligns protein sequence representations with structured biological attributes. Across intra-species benchmark PPI datasets, HIPPO improves the average micro-F1 by 2.9% compared with the best baseline across the evaluated splits. In the host–pathogen interaction benchmark, HIPPO achieves the highest AUROC under the standard split (0.731) and the second-best AUPRC (0.332). Under leave-one-virus-family-out evaluation, HIPPO obtains the best AUROC on Papillomaviridae (0.603) and Retroviridae (0.612), while also showing family-dependent transfer behavior. Ablation experiments support the contribution of hierarchical feature integration, and attention-based residue attribution provides preliminary evidence that the learned representations highlight interface-related residues. Together, these results suggest that structured biological knowledge can improve representation learning for PPI prediction across diverse and imbalanced datasets.

## 1. Introduction

Protein–protein interactions (PPIs) represent one of the most fundamental molecular processes in living systems and are central to cellular regulation and disease-related mechanisms [1,2,3]. Historically, PPIs have been investigated using experimental techniques such as yeast two-hybrid systems [4], co-immunoprecipitation assays [5], affinity purification or pull-down-based assays [6], chemical cross-linking [7], and proximity-based labeling [8]. These approaches have provided essential information for mapping molecular interaction networks, but they differ in throughput, sensitivity, and the types of interactions they preferentially capture [9]. However, the growing need to map vast interactomes, coupled with the prohibitively high costs and labor-intensive nature of experimental approaches, has rendered large-scale wet-lab characterization of PPIs increasingly impractical. To address this, computational strategies have gained prominence as scalable and cost-efficient alternatives, enabling systematic prediction and mapping of PPIs [10].

Recently, artificial intelligence (AI) and deep learning in particular have become widely used in protein representation learning and computational biology [11,12,13]. From sequence-based models to structure-aware neural architectures, AI-driven approaches have demonstrated strong performance in a wide range of tasks, including protein structure prediction [14], functional annotation [15], and interaction inference [16]. A particularly promising class of methods in this domain is contrastive learning [17,18,19], which belongs to the broader family of self-supervised learning techniques. By learning to discriminate between semantically similar and dissimilar pairs, contrastive models are capable of uncovering informative and generalizable representations, even in the absence of explicit labels. This paradigm has been successfully applied in computer vision and vision-language representation learning [17,18], and its adaptation to biological data has recently gained traction [20].

In the context of PPI prediction, contrastive learning offers a compelling framework for capturing relationships between protein sequences and associated biological features. By training models to pull together representations of interacting protein pairs while pushing apart non-interacting ones, contrastive learning naturally aligns with the relational nature of PPI prediction. Moreover, it enables the integration of diverse biological modalities—such as amino acid sequences, structural domains, and functional annotations—into a unified representation space. However, despite these advantages, several representation-learning approaches for PPI prediction encode protein information without explicitly modeling hierarchical relationships among protein families, domains, and functional annotations [16,21]. Proteins are classified into families, superfamilies, and domains [22,23,24,25] and are annotated with hierarchical ontologies such as Gene Ontology (GO) [26], reflecting multiple levels of biological specificity and generalization. Representing these structured relationships may provide additional biological context for PPI prediction and model interpretation.

Structure-based approaches are widely applied in general protein modeling due to their ability to capture spatial and functional features of well-folded domains [14,27]. However, in the context of protein–protein interaction (PPI) prediction, their utility can be limited by the prevalence of intrinsically disordered regions (IDRs), which do not form stable tertiary structures but instead exist as dynamic ensembles in solution [28]. Sequence-based frameworks therefore provide a complementary route for modeling PPIs, especially when reliable complex structures are unavailable or when interactions involve both ordered and disordered protein regions.

In this work, we propose a hierarchical contrastive learning framework tailored for protein–protein interaction prediction. Our method integrates protein sequence representations with structured biological knowledge through a multi-tiered contrastive objective. The framework uses hierarchical contrastive losses to encourage protein representations to reflect relationships among protein families, domains, and functional annotations. We evaluate the framework on benchmark PPI datasets under intra-species and cross-species settings, and further examine host–pathogen interaction prediction as a challenging inter-species case study. Ablation analyses are used to assess the contribution of hierarchical feature integration, and attention-based residue attribution is used to examine whether attention-highlighted residues overlap with annotated interface-related regions.

By combining contrastive learning with structured biological attributes, this work provides a framework for incorporating hierarchical biological knowledge into PPI representation learning and evaluates its behavior across multiple benchmark settings.

## 2. Results

### 2.1. HIPPO Introduces Joint Protein Sequences and Hierarchical Relationship Learning of Proteins and the PPI Network

Existing models [16,29,30] primarily learn the protein interactome based on sequence or structural information. While these models are effective in capturing secondary and tertiary structural features, they often fall short in explicitly modeling diverse interaction properties and their relationships among proteins, such as functions, post-translational modifications (PTMs), and binding domains. To address this limitation, we propose a hierarchical multimodal pretraining framework designed to extract rich, structured protein representations for PPI prediction. This framework integrates heterogeneous information about proteins from different sources and models both molecular content and biological context. Specifically, interacting proteins are first represented by their linear amino acid sequences, enriched with biophysical descriptors. Then, the interactions between proteins are captured using a protein interaction graph, in which proteins serve as nodes and their interactions as edges.

To comprehensively model protein–protein interactions, our framework is explicitly designed to integrate both hierarchical biological relationships and network-level interaction patterns. Sequence-derived features are obtained via a Protein Language Model (PLM), while an Annotation Language Model (ALM) encodes non-hierarchical annotations. Hierarchical information—including protein family and domain structures—is leveraged as supervisory labels in hierarchical contrastive learning, guiding the representation space to reflect biologically meaningful hierarchies. Simultaneously, a Graph Neural Network (GNN) is employed to model the global structure of the PPI network, ensuring that the learned embeddings capture not only local sequence and annotation information but also topological dependencies among interacting proteins. Together, these components enable our model to preserve and exploit both hierarchical protein relationships and the structural complexity of PPI networks.

The architecture is explicitly designed to integrate hierarchical biological knowledge with the topological structure of protein–protein interaction (PPI) networks. It operates in two primary stages: (1) feature extraction and (2) hierarchical and network-aware representation learning.

In the feature extraction stage, two parallel encoding streams are employed: a sequence encoder processes amino acid sequences, while an annotation encoder encodes non-hierarchical protein functions as binary vectors. Both encoders utilize six transformer-like blocks with skip connections and layer normalization to enhance expressiveness and stability. Local features are refined through a combination of 1D—both narrow and wide—which together capture context ranging from short- to long-range dependencies. This setup yields rich representations that encompass both sequence-level and annotation-derived characteristics.

To incorporate biological hierarchy, we introduce a hierarchical multi-label contrastive learning objective. Hierarchical annotations—such as family and domain relationships—are used as supervisory signals, guiding the model to structure the embedding space according to known biological hierarchies. This encourages proteins with similar hierarchical attributes to cluster together, thereby embedding functional relationships directly into the latent space.

**Figure 1:**
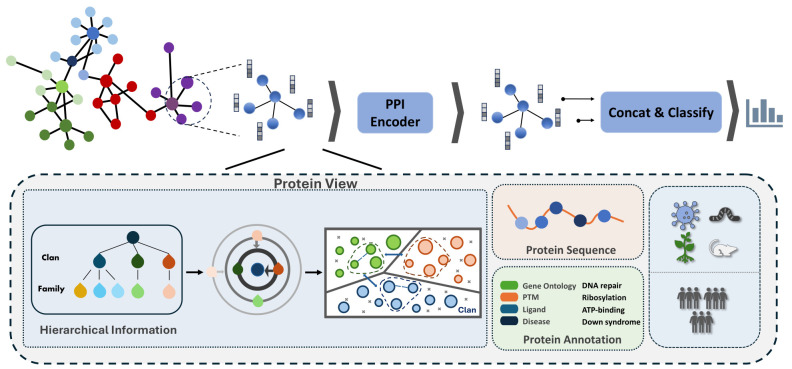
Overview of the HIPPO architecture. The proposed framework integrates protein sequences and hierarchical attributes to learn biologically meaningful representations for PPI prediction. In the top panel (PPI network), proteins are modeled as nodes, interactions are represented as edges, and the representations obtained from the bottom protein view are used as node features. Protein embeddings are refined through a trainable PPI encoder. For each query pair, the updated node representations are concatenated and passed through a classifier to predict protein–protein interactions. In the bottom panel (Protein View), protein sequences are aligned with functional annotations, including Gene Ontology terms, post-translational modifications (PTMs), ligand interactions, and disease associations. Hierarchical attributes, such as protein family relationships, are structured into a tree and embedded via contrastive learning that enforces locality in the representation space. Proteins with similar evolutionary traces are encouraged to cluster together, preserving hierarchical relationships in the latent space.

For PPI network modeling, the unified protein embeddings are used as initial node features in a PPI graph, where interactions are represented as edges. To capture complex topological patterns, we employ a Graph Isomorphism Network (GIN) consisting of three recursive blocks, each with a GIN layer, ReLU activation, and Batch Normalization. This enables the model to aggregate contextual information from neighboring proteins and encode network-dependent features critical for interaction prediction. In the final prediction stage, embeddings from two candidate proteins are concatenated and passed through a Multi-Layer Perceptron (MLP) classifier. This design allows hierarchical protein relationships and PPI network context to jointly contribute to the final interaction prediction. Figure 1 provides an overview of the HIPPO architecture.

### 2.2. HIPPO Achieves Strong Performance in Intra-Species PPI Prediction

To validate the predictive power of the proposed model, we benchmarked its performance against leading methods from three perspectives: (1) overall predictive accuracy on three human datasets using depth-first search (DFS), breadth-first search (BFS), and random split strategies; (2) generalization across varying levels of prediction difficulty; and (3) classification accuracy across five distinct PPI types.

**Figure 2:**
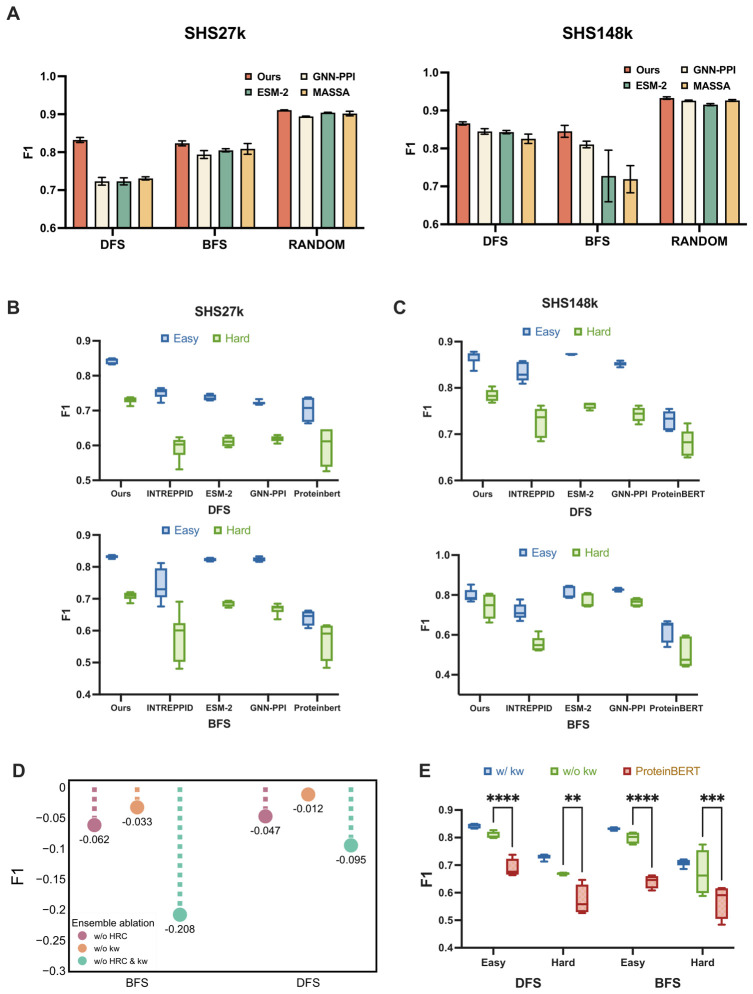
Performance of HIPPO in predicting PPIs. (**A**) Comparison of model performance across human PPI datasets. Bar plots show $F_{1}$ scores of four models (Ours, GNN-PPI, ESM-2, and MASSA) evaluated on the SHS27k and SHS148k datasets using depth-first search (DFS), breadth-first search (BFS), and random split strategies. HIPPO achieves the highest or near-highest performance across the evaluated settings. (**B**) Comparative analysis across prediction difficulty levels in the SHS27k dataset. PPIs are categorized as easy (at least one interacting protein appears in the training set) or hard (both proteins are unseen during training). Box plots compare $F_{1}$ scores across difficulty subsets under DFS and BFS split settings. (**C**) Comparative analysis across prediction difficulty levels in the SHS148k dataset. (**D**) Change in performance on SHS27k and SHS148k after excluding hierarchical attributes (HRC) and keyword annotations (kw). Scatter plots display the relative performance drop ($\Delta F_{1}$) resulting from ablating key components; each point is computed from the mean $F_{1}$ across repeated runs. (**E**) Comparison of the full protein-view encoder with keyword annotations (“w/kw”), without keyword annotations (“w/o kw”), and the ProteinBERT-structured encoder without hierarchical contrastive learning. ProteinBERT is included here as the architecture reference for the protein-view encoder when the hierarchical objective is not applied. Statistical significance is indicated by asterisks: **** (p<0.0001), *** (p<0.001), and ** (p<0.01).

For a rigorous comparison, we froze the protein encoder to ensure consistent node feature extraction for the PPI graph and performed PPI type prediction using a shared classifier layer for all models. Bar plots in Figure 2A summarize the F1 scores of four models: our approach, GNN-PPI, ESM-2, and MASSA on the SHS27k and SHS148k datasets, each evaluated under three different splitting strategies. The proposed method consistently achieves superior performance, improving the average micro-F1 by 2.9% compared to the best baseline across all splits. Notably, under the DFS split, our model outperforms GNN-PPI by 10.9% on the SHS27k dataset, where partitioning is less favorable for learning from previously seen proteins. In contrast, the performance gap narrows considerably under the random split, with GNN-PPI slightly outperforming our model by 1.7%, indicating that both models are comparably effective when the data distribution is less stringent. All models demonstrate higher F1 scores on SHS148k compared to SHS27k, reflecting the greater redundancy and coverage of protein interactions in the larger dataset. Under the DFS split, our model surpasses GNN-PPI by 2.1%. This advantage persists and becomes more pronounced under the BFS split, where our model has a 3.5% improvement. Notably, model performance under the BFS split exhibits substantially higher variance across all methods. Under random split, the performance gap between methods is minimized, consistent with the less challenging nature of random partitioning. Overall, these results indicate that hierarchical feature integration improves predictive accuracy across the evaluated intra-species PPI benchmarks.

To further assess generalization, we stratified protein–protein pairs into “easy” and “hard” categories: in the former, at least one interacting protein is seen during training, while in the latter, both proteins are unseen. As shown in Figure 2B, all models achieve comparable F1 scores on easy pairs across both datasets and split strategies, indicating similar predictive capacity when at least partial training information is available. However, the differences between models become much more pronounced on hard pairs. While all methods experience a performance drop in this scenario, our model consistently outperforms the baselines and exhibits lower prediction variance. This advantage is particularly notable on the SHS27k dataset, where the gap between our method and others widens substantially for hard cases.

### 2.3. Modeling Hierarchical Relationships Among Proteins Improves Performance

**Figure 3:**
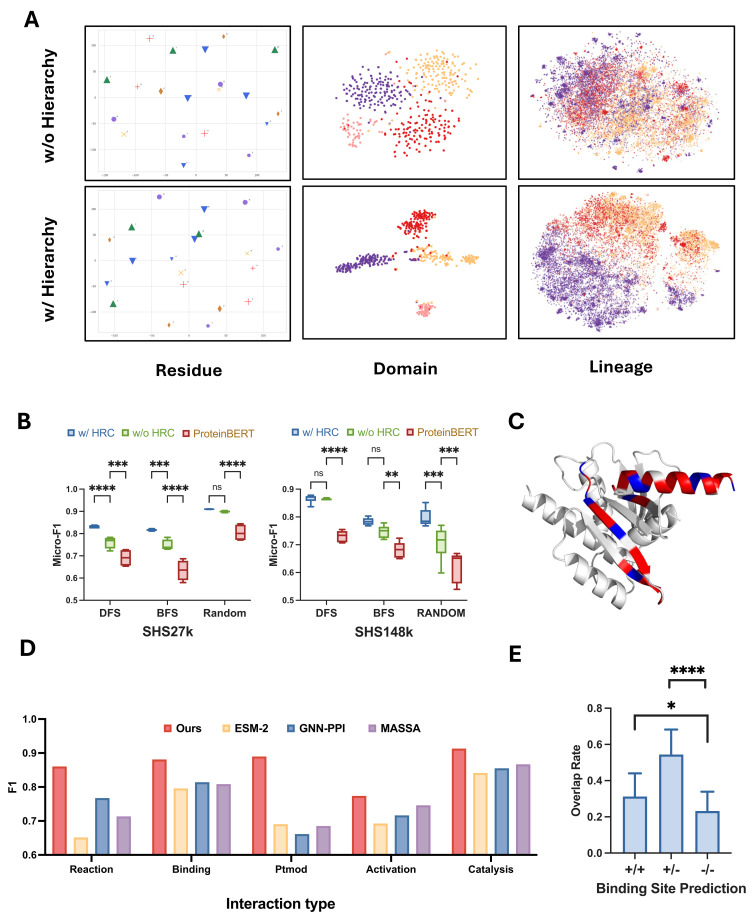
Protein embeddings and attention analysis for PPI prediction. (**A**) Protein embeddings at residue, domain, and lineage levels with or without hierarchical pre-training. The comparative visualization illustrates improved clustering and distinct separation patterns in the lower row (w/Hierarchy), demonstrating the enhanced representational capability achieved by incorporating hierarchical constraints during training. Residue-level embeddings are colored and shaped by physicochemical properties, domain-level embeddings distinguish representative protein domains, and lineage-level embeddings visualize global protein embeddings from the Beta-lactamase family PF00144 from Pfam. (**B**) Micro-F1 comparison on SHS27k and SHS148k after removing hierarchical relationship constraints (w/o HRC), compared with the full model (w/HRC) and ProteinBERT. (**C**) Residue attribution based on attention in the chicken Spc24–Spc25 globular domain (PDB ID: 3VZ9). Red marks highlight residues that overlap with annotated interface-related residues, blue marks highlight residues outside the annotated set, and white marks other protein regions. (**D**) F1 scores across five PPI types. Bar plots show F1 scores of four models (Ours, ESM-2, GNN-PPI, and MASSA) across Reaction, Binding, PTMod, Activation, and Catalysis under DFS, BFS, and random split strategies on the SHS27k dataset. (**E**) Overlap between attention-highlighted residues and annotated interface-related residues. Statistical significance is indicated as follows: ns, not significant; * p<0.05; ** p<0.01; *** p<0.001; **** p<0.0001.

As shown in Figure 2D and Figure 3B, the removal of hierarchical relationships among protein properties resulted in a marked decrease in predictive accuracy across all intra-species datasets. The F1 scores dropped by up to 15% when hierarchical labels were omitted, underscoring the significant contribution of structured biological information. Notably, the performance degradation was most pronounced on “hard” test pairs, where both interacting proteins were unseen during training, suggesting that hierarchical modeling is particularly beneficial for generalizing to novel or under-annotated proteins.

Further analysis revealed that integrating hierarchical relationships facilitates the learning of protein representations that more effectively capture functional similarities and evolutionary relationships. This structural prior enables the model to cluster proteins with shared functional characteristics more closely in the embedding space, thereby improving the discrimination between interacting and non-interacting pairs.

To evaluate whether hierarchical supervision helps the model capture biologically meaningful structure, we visualized the learned protein embeddings at three levels-residue, domain, and lineage, with and without hierarchical constraints, as shown in Figure 3A. In the absence of hierarchical supervision, the resulting representations show limited organization, with protein points dispersed and clusters poorly defined across all annotation levels. When hierarchical relationships are incorporated, clear and compact clustering emerges at the domain and lineage levels, indicating that the model is able to learn functionally relevant structure by leveraging hierarchical information.

Furthermore, we examined the specific contribution of non-hierarchical information by excluding non-hierarchical annotations from the model. As shown in Figure 2D, removing non-hierarchical information and relying solely on hierarchical annotations led to a noticeable decrease in intra-species prediction accuracy. Visualizations of the learned embeddings at both residue and lineage levels reveal that, with only family supervision, the representations remain scattered and lack clear clustering. As shown in Figure 3E, this ablation produced higher overlap between attention-highlighted residues and annotated interface-related residues. This result suggests that hierarchical annotations can strengthen residue-level attribution around interface-related regions, although non-hierarchical annotations remain valuable for general PPI prediction performance.

Collectively, these results demonstrate that modeling hierarchical relationships among protein properties is critical for enhancing predictive performance, especially in challenging prediction scenarios involving unfamiliar proteins.

### 2.4. HIPPO Extends to Cross-Species PPI Prediction with Leading Performance

Cross-species protein–protein interaction (PPI) prediction addresses the challenge of inferring interactions in less-studied species by leveraging knowledge learned from well-studied organisms, such as humans. In this section, we evaluate whether the learned representations support transfer across species boundaries under the benchmark settings considered in this study. Specifically, we evaluate PPI prediction in six species after training on human datasets: *Escherichia coli*, *Saccharomyces cerevisiae*, *Mus musculus*, *Caenorhabditis elegans*, *Arabidopsis thaliana*, and *Drosophila melanogaster*.

**Figure 4:**
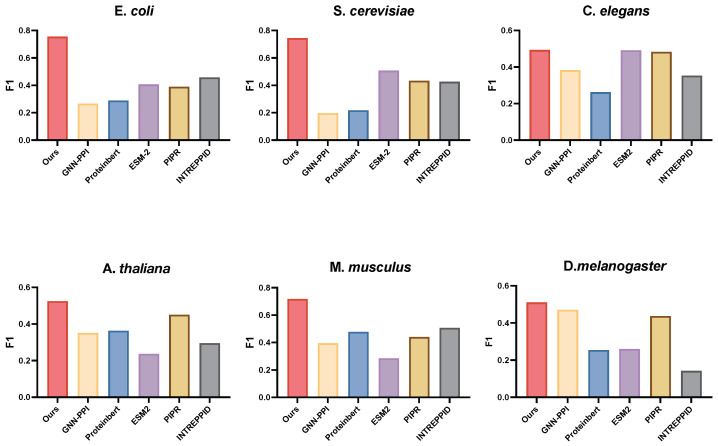
Performance comparison of models in predicting cross-species protein–protein interactions (PPIs). Bar plots depict Micro-F1 scores across six cross-species datasets: *Escherichia coli* (*E. coli*), *Saccharomyces cerevisiae* (*S. cerevisiae*), *Caenorhabditis elegans* (*C. elegans*), *Mus musculus* (*M. musculus*), *Arabidopsis thaliana* (*A. thaliana*), and *Drosophila melanogaster* (*D. melanogaster*). The proposed method (Ours) is benchmarked against five state-of-the-art models: GNN-PPI, ProteinBERT, ESM-2, PIPR, and INTREPPID.

As summarized in Figure 4, our model achieves top or near-top performance compared with existing methods, including GNN-PPI, ProteinBERT, ESM-2, PIPR, and INTREPPID, across the evaluated cross-species datasets. These results suggest that the hierarchical, multimodal framework can learn transferable protein representations under the tested cross-species settings.

The performance is consistent with the possibility that the learned representations capture conserved functional features and interaction patterns shared across the evaluated organisms. These findings support further investigation of hierarchy-aware representation learning for cross-species PPI prediction.

### 2.5. Residue Attribution Based on Attention Highlights Interface-Related Regions in Interaction Complexes

To examine how hierarchical pretraining affects residue-level model attribution, we analyzed attention weights extracted from the protein-view encoder. Annotated protein–protein binding-site labels were obtained from the EDLMPPI dataset described by Hou et al. [31], and the attention-based attribution procedure follows the protein language model interpretation framework of Vig et al. [32]. In this analysis, attention-highlighted residues are compared with annotated interface-related residues to assess overlap-based agreement.

Figure 3C provides a structural visualization based on the crystal structure of the chicken Spc24-Spc25 globular domain (PDB ID: 3vz9). Residues shown in red indicate attention-highlighted residues that overlap with annotated interface-related residues, whereas residues shown in blue indicate highlighted residues outside the annotated set. Incorporating hierarchical contrastive learning increases the overlap score from 0.40625 to 0.71875 in this representative complex, suggesting that hierarchical supervision makes attention-derived residue attribution more concentrated around annotated interface-related regions.

In Figure 3E, we further summarize overlap-based attribution results across 35 protein complexes by comparing three model configurations: hierarchical information combined with flat annotations (+/+), hierarchical information without flat annotations (+/−), and neither hierarchical nor flat annotations (−/−). Models incorporating hierarchical annotations (+/+ and +/−) show higher overlap with annotated interface-related residues than the annotation-free model (−/−), supporting the contribution of hierarchical supervision to residue-level attribution.

Collectively, this analysis indicates that hierarchical pretraining improves the agreement between attention-highlighted residues and annotated interface-related regions. These results are interpreted as residue-level model-attribution evidence for the learned protein representations.

### 2.6. Case Study: HIPPO Transfers to Host–Pathogen Interaction Prediction

The preceding experiments demonstrate that HIPPO learns protein representations with strong cross-species generalization ability from protein sequences and hierarchical biological attributes. A natural follow-up question is whether these PPI-oriented representations can transfer to a related downstream task that is not observed during pretraining, without updating the encoder parameters. To investigate this question, we use host–pathogen interaction (HPI) prediction as a case study.

Unlike conventional intra-species or cross-species PPI prediction, HPI prediction requires the model to predict interactions between proteins from a host and a pathogen. In this study, we focus on interactions between human host proteins and viral proteins. This setting introduces a substantial distribution shift. Human and viral proteins are evolutionarily distant, and viral proteins are often sparsely annotated. More importantly, HIPPO is pretrained on a relatively small protein dataset primarily composed of human and a limited number of eukaryotic proteins, without any viral protein sequences. HPI prediction, therefore, provides a challenging setting for evaluating whether PPI-oriented protein representations can transfer to host–virus interaction prediction.

The construction of the HPI benchmark, the negative sampling protocol, the standard split, the LOVO split, and the downstream evaluation settings are described in Section 4.1.

#### 2.6.1. HIPPO Shows Transfer Performance Under the Standard HPI Split

**Figure 5:**
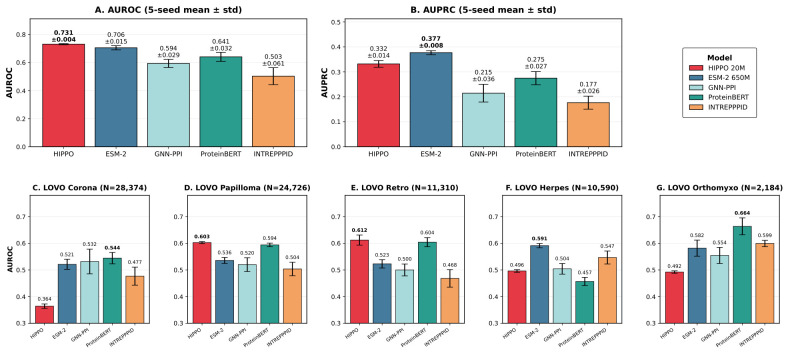
Host–pathogen interaction prediction case study. (**A**,**B**) AUROC and AUPRC under the standard HPI split, reported as the mean ± standard deviation over five independent runs. (**C**–**G**) Leave-one-virus-family-out (LOVO) AUROC results for Coronaviridae, Papillomaviridae, Retroviridae, Herpesviridae, and Orthomyxoviridae. HIPPO is compared against ESM-2, GNN-PPI, ProteinBERT, and INTREPPID using a unified downstream prediction setting.

Table 1 summarizes the performance under the standard HPI split, and Figure 5A,B visualizes the corresponding AUROC and AUPRC results. To maintain consistency with the PPI benchmark comparisons, HIPPO is evaluated against ESM-2, GNN-PPI, ProteinBERT, and INTREPPID using the same downstream prediction setting. HIPPO achieves the highest AUROC of 0.731±0.004, followed by ESM-2 (0.706±0.015), ProteinBERT (0.641±0.032), GNN-PPI (0.594±0.029), and INTREPPID (0.503±0.061).

For AUPRC, ESM-2 obtains the highest value (0.377±0.008), while HIPPO ranks second with 0.332±0.014. ESM-2 also obtains the highest macro-F1 (0.640±0.007), followed by HIPPO (0.601±0.020). These results indicate that HIPPO retains transfer performance under the standard HPI split, although AUPRC remains challenging because the benchmark is highly class-imbalanced.

These results show that a compact encoder trained for PPI representation learning can retain transferability to HPI prediction under the standard split. The comparison also suggests that different evaluation metrics emphasize different aspects of transfer performance, with HIPPO ranking first by AUROC and ESM-2 ranking first by AUPRC.

#### 2.6.2. LOVO Evaluation Shows Family-Dependent Transfer to Unseen Viral Families

The standard HPI split evaluates generalization to unseen interaction pairs under the same overall data distribution. To further assess whether the learned representations can extrapolate to viral groups that are entirely absent from downstream training, we adopt a more stringent leave-one-virus-family-out (LOVO) protocol. In each round, all interactions associated with one viral family are held out as the test set, while interactions from the remaining viral families are used to train the downstream prediction head. The held-out family is not used for either training or validation.

Because AUROC estimates for very small viral families are dominated by sampling noise, we focus the LOVO analysis on the five largest families: Coronaviridae, Papillomaviridae, Retroviridae, Herpesviridae, and Orthomyxoviridae. Compared with the standard split, LOVO introduces a substantially stronger distribution shift. This setting is particularly challenging for HIPPO: the encoder is pretrained without any viral proteins, and the held-out viral family is also completely absent from downstream training.

As shown in Table 2 and Figure 5C–G, the encoders exhibit distinct family-specific performance patterns. HIPPO achieves the highest AUROC on Papillomaviridae (0.603) and Retroviridae (0.612). ProteinBERT performs best on Coronaviridae (0.544) and Orthomyxoviridae (0.664), while ESM-2 performs best on Herpesviridae (0.591). HIPPO performs less well on Coronaviridae, Herpesviridae, and Orthomyxoviridae, indicating that its transfer behavior depends strongly on the held-out viral family.

The LOVO results highlight the difficulty of transferring HPI predictors to completely unseen viral families. HIPPO shows family-dependent transfer behavior, with the best results on Papillomaviridae and Retroviridae but weaker performance on Coronaviridae, Herpesviridae, and Orthomyxoviridae. These findings indicate that the PPI-oriented representations learned by HIPPO retain partial transferability to the HPI setting, while also showing clear limitations under strong viral-family distribution shifts.

#### 2.6.3. Summary

Overall, this case study shows that HIPPO achieves the highest AUROC and the second-best AUPRC under the standard HPI split when compared with ESM-2, GNN-PPI, ProteinBERT, and INTREPPID. Under the more stringent LOVO protocol, HIPPO is affected by the substantial distribution shift introduced by completely unseen viral families, ranking first on Papillomaviridae and Retroviridae but performing less well on other held-out families.

These results support the use of HPI prediction as a challenging transfer case study and indicate that family-level distribution shifts remain a major difficulty for human–virus interaction prediction.

## 3. Discussion

In this study, we introduced HIPPO as a hierarchical contrastive learning framework for protein–protein interaction (PPI) prediction across intra-species and cross-species settings. The framework integrates protein sequence information with curated biological attributes, including family/domain annotations and functional keywords, to examine how structured biological knowledge contributes to PPI representation learning.

The benchmark results suggest that hierarchical feature integration improves PPI prediction and supports transfer across several cross-species evaluation settings. The host–pathogen interaction (HPI) case study further provides a more challenging inter-species evaluation scenario. In particular, the leave-one-virus-family-out (LOVO) protocol introduces a strong distribution shift because the held-out viral family is absent from downstream training. HIPPO shows transfer performance under the standard HPI split, but its performance varies substantially across viral families under LOVO, indicating that human–virus interaction prediction remains difficult when viral proteins are taxonomically or functionally distant from the training data.

Several limitations should be considered when interpreting these results. First, the effectiveness of hierarchy-aware learning depends on the coverage and quality of available annotations, which may be uneven across organisms and protein families. Second, the residue-level analysis is an attention-based model-attribution analysis. The highlighted residues provide preliminary attribution evidence based on overlap with interface-related regions. Future benchmark studies may further compare HIPPO with additional protein-specific hierarchical representation learning methods. Future work will focus on improved modeling of context-specific interactions.

## 4. Materials and Methods

### 4.1. Datasets and Benchmark Splits

During the pretraining stage, we employed the Swiss-Prot dataset from UniProtKB [33,34] due to its extensive protein data coverage and high-quality, manually curated annotations. Protein attributes include various protein families and clans accessible through the Pfam database [24,25], and annotations curated within the UniProtKB Keywords section. The keywords section incorporates controlled vocabulary terms manually annotated to include Gene Ontology (GO) terms, disease associations, protein domains, ligands, and post-translational modifications (PTMs).

During the protein–protein interaction (PPI) prediction stage, we utilized three datasets derived from STRING [10]: the full STRING dataset, SHS148k, and SHS27k. The STRING dataset contains 1,048,575 interactions among 16,073 proteins. SHS27k and SHS148k were created by selecting proteins longer than 50 amino acids with less than 40% sequence identity to form more challenging subsets. SHS27k comprises 63,408 interactions among 1690 proteins, while SHS148k includes 36,902 interactions among 5189 proteins. Three partition methods—Random, Breadth-First Search (BFS), and Depth-First Search (DFS)—were employed for dataset splitting [16]. We used these benchmark splits to evaluate both standard within-distribution prediction and more stringent generalization to proteins that were absent from the training set.

For the host–pathogen interaction (HPI) benchmark, we integrated nine public HPI databases and molecular-interaction resources, including HVIDB-2025, HVIDB, PHISTO, HPIDB [35], IntAct [36], VirHostNet [37,38], VirusMentha, BioGRID, and EBI-GOA non-IntAct. After deduplication, the integrated dataset contained 13,224 experimentally supported human–virus interactions involving 4894 human host proteins and 264 viral proteins from 10 viral families. The interaction evidence was supported by multiple sources: 66.5% of positive interactions were reported by at least two databases, and 61.4% were reported by at least three databases.

Negative HPI examples were uniformly sampled from human–virus protein pairs that did not occur among the known positive interactions. Because unobserved interactions are not necessarily experimentally confirmed negatives, this sampling strategy may introduce false-negative bias; we therefore interpret the HPI results as benchmark performance under a commonly used unobserved-pair negative sampling assumption. A positive-to-negative ratio of 1:5 was maintained in all HPI experimental splits. To reduce sequence-level leakage, all proteins were clustered using CD-HIT [39] at a 60% sequence identity threshold and assigned to mutually exclusive training, validation, and test sets. Protein pairs crossing different splits were removed. The resulting benchmark contained 42,690 interaction pairs, corresponding to approximately 54% of the complete candidate dataset. Table 3 summarizes the numbers of positive, negative, and total interaction pairs in the standard HPI split.

For the standard HPI split, frozen protein encoders were evaluated using a shared shallow downstream prediction head. For each host–virus protein pair (h,v), the frozen embeddings of the two proteins were concatenated with Pfam-derived interaction prior features and passed to a two-layer multilayer perceptron to predict the interaction probability. All encoders used the same downstream architecture, optimization procedure, and early-stopping criterion. We also evaluated leave-one-virus-family-out (LOVO) generalization by holding out all interactions associated with one viral family as the test set and training the downstream head on interactions from the remaining viral families. Because the HPI benchmark is class-imbalanced, AUROC and AUPRC were used as the primary metrics.

### 4.2. Protein Hierarchy Construction and Pair Sampling

Protein hierarchy is constructed from Pfam family and clan annotations. In this hierarchy, Pfam clans represent broader evolutionary groups, and Pfam families represent more specific protein groups within each clan. The resulting clan and family annotations are used for hierarchy-aware protein contrastive learning. For an anchor protein, proteins sharing at least one Pfam family with the anchor are defined as family-level positives. Proteins sharing at least one Pfam clan but no Pfam family with the anchor are defined as clan-level positives. Proteins sharing neither Pfam family nor Pfam clan membership with the anchor are treated as negatives within the mini-batch. The family and clan terms are combined with level-specific weights so that the embedding space can preserve graded evolutionary relatedness. Our framework comprises 6329 distinct families and 621 clans, forming a clan-family hierarchy that reflects evolutionary relationships, sequence similarity, and structural homology [25].

### 4.3. Model Architecture

HIPPO denotes a two-view framework with a shared protein-view encoder and benchmark-specific PPI network-view predictors. In the protein view, the protein encoder is pretrained on Swiss-Prot using hierarchical contrastive learning over protein-level biological attributes. This pretrained protein-view encoder is kept fixed in all downstream benchmark experiments. In the PPI network view, a dataset-specific interaction prediction module is trained on each benchmark dataset. Across the SHS27k, SHS148k, and HPI experiments, HIPPO therefore uses a shared frozen protein-view encoder followed by benchmark-specific PPI network-view predictors trained according to their respective dataset protocols.

Given a protein set $P=\{p_{0},p_{1},\ldots ,p_{n}\}$ and a corresponding set of protein–protein interactions (PPIs)

$$X=\{x_{ij}=\left\{p_{i},p_{j}\right\}|i\neq j,p_{i},p_{j}\in P\},$$

we define the PPI label space as

$$L=\{l_{0},l_{1},\ldots ,l_{n}\},$$

where each interaction $x_{ij}$ is associated with a label set $y_{ij}\subseteq L$, indicating multiple potential interaction types. The full dataset is denoted as

$$D=\{\left(x_{ij},y_{ij}\right)|x_{ij}\in X\},$$

and can be naturally represented as a graph G=(P,X), where proteins are nodes and interactions are labeled edges.

To incorporate functional and evolutionary context, we encode each protein into a hierarchical embedding informed by family- and clan-level annotations. These representations capture evolutionary lineage and structural similarity, which are essential for understanding protein interaction mechanisms.

We formulate the multi-label PPI task as learning a prediction function

$$F:x_{ij}\to {\hat{y}}_{ij},$$

using a training subset $X_{\mathrm{train}}\subseteq X$, with evaluation performed on a disjoint test set $X_{\mathrm{test}}$, satisfying $X_{\mathrm{train}}\cup X_{\mathrm{test}}=X$.

To implement this, we adopt the Graph Isomorphism Network (GIN) [40] as the backbone encoder. GIN aggregates neighborhood information from each protein node to produce discriminative embeddings. For each protein pair $x_{ij}$, the embeddings $g_{p_{i}}$ and $g_{p_{j}}$ are combined using a dot product, followed by a fully connected (FC) layer for final prediction:

$${\hat{y}}_{ij}=\mathrm{FC}\left(g_{p_{i}}\cdot g_{p_{j}}\right).$$

The model is trained using a binary cross-entropy loss over all interaction types:

$$L=\sum_{k=0}^{n}\sum_{x_{ij}\in X_{\mathrm{train}}}\left(-y_{ij}^{k}\log{\hat{y}}_{ij}^{k}-\left(1-y_{ij}^{k}\right)\log\left(1-{\hat{y}}_{ij}^{k}\right)\right),$$

where $y_{ij}^{k}$ and ${\hat{y}}_{ij}^{k}$ denote the ground truth and predicted probability of the *k*-th interaction type for the protein pair $x_{ij}$.

### 4.4. Hierarchical Contrastive Learning Objective

To enable effective downstream prediction of protein–protein interactions, the protein-view encoder is pretrained with two complementary contrastive components. Pfam family and clan labels are used for hierarchy-aware protein contrastive learning, while functional annotations are incorporated through sequence–annotation contrastive alignment.

#### 4.4.1. Hierarchical Relationship Contrastive Learning

Pfam family and clan annotations provide the hierarchy used in this component. Drawing on hierarchical multi-label contrastive learning [41], HIPPO defines positive protein pairs according to shared family or clan membership. Same-family proteins are treated as closer positives, and different-family proteins from the same clan are treated as broader-level positives. In-batch proteins with no shared family or clan membership are used as negatives. For a mini-batch B, let $h_{i}$ denote the normalized protein embedding of anchor protein *i*. Let $F_{i}$ and $C_{i}$ denote the sets of Pfam families and Pfam clans assigned to protein *i*, respectively. The family-level positive set is defined as $P_{F}\left(i\right)=\left\{j\in B\setminus i:F_{i}\cap F_{j}\neq \emptyset \right\}$. The clan-level positive set is defined as $P_{C}\left(i\right)=\left\{j\in B\setminus i:C_{i}\cap C_{j}\neq \emptyset ,F_{i}\cap F_{j}=\emptyset \right\}$. The in-batch negative set is $N\left(i\right)=\left\{j\in B\setminus i:F_{i}\cap F_{j}=\emptyset ,C_{i}\cap C_{j}=\emptyset \right\}$. For each hierarchy level l∈{F,C}, where *F* denotes family and *C* denotes clan, let $B_{l}=\left\{i\in B:\right|P_{l}\left(i\right)\left|>0\right\}$ denote anchors with at least one positive sample at level *l*. The level-specific supervised contrastive loss is

(1) $$L_{l}=-\frac{1}{|B_{l}|}\sum_{i\in B_{l}}\frac{1}{|P_{l}\left(i\right)|}\sum_{p\in P_{l}\left(i\right)}\log\frac{\exp(h_{i}^{⊤}h_{p}/\tau_{h})}{\sum_{a\in B\setminus i}\exp\left(h_{i}^{⊤}h_{a}/\tau_{h}\right)},$$

where $P_{l}\left(i\right)$ is the positive set for level *l*. The denominator includes all non-anchor proteins in the mini-batch, including the level-specific positives and in-batch negatives. Anchors with no positive samples at level *l* are omitted from the corresponding summation. The hierarchy-aware protein contrastive loss is then

(2) $$L_{\mathrm{HC}}=\lambda_{F}L_{F}+\lambda_{C}L_{C},$$

where $\tau_{h}$ is the temperature parameter, and $\lambda_{F}$ and $\lambda_{C}$ control the relative contributions of family-level and clan-level positives. These weights allow the objective to encode graded similarity between family-level and clan-level relationships.

#### 4.4.2. Multimodal Sequence–Annotation Alignment

Functional annotations are incorporated through a CLIP-style sequence–annotation contrastive objective. This component aligns each protein sequence embedding with its corresponding annotation-derived embedding and separates it from mismatched annotations in the same mini-batch. Specifically, we adapt the sequence and annotation encoding modules from ProteinBERT [42] and perform end-to-end pre-training. Protein sequences are encoded using a protein language model (PLM), producing sequence representations ${\left\{s_{i}\right\}}_{i=1}^{N}$ for a batch of *N* proteins. In parallel, the annotation encoder processes curated functional annotation features and produces annotation representations ${\left\{a_{i}\right\}}_{i=1}^{N}$. To maximize the agreement between matching sequence–annotation pairs while discouraging spurious alignments, we employ a symmetric InfoNCE loss [19]:

(3) $$L_{\mathrm{SAC}}=-\frac{1}{2N}\sum_{i=1}^{N}\left(\log\frac{\exp(s_{i}^{⊤}a_{i}/\tau_{a})}{\sum_{j=1}^{N}\exp\left(s_{i}^{⊤}a_{j}/\tau_{a}\right)}+\log\frac{\exp(a_{i}^{⊤}s_{i}/\tau_{a})}{\sum_{j=1}^{N}\exp\left(a_{i}^{⊤}s_{j}/\tau_{a}\right)}\right),$$

where $\tau_{a}$ is the sequence–annotation contrastive temperature. To further distinguish between truly corresponding (positive) and mismatched (negative) sequence–annotation pairs, we introduce a Sequence–Annotation Matching (SAM) loss using focal loss:

(4) $$L_{\mathrm{SAM}}=-\frac{1}{N}\sum_{i=1}^{N}\left(\alpha {\left(1-p_{i}\right)}^{\gamma }\log\left(p_{i}\right)\cdot y_{i}^{\mathrm{SAM}}+\left(1-\alpha \right)p_{i}^{\gamma }\log\left(1-p_{i}\right)\cdot \left(1-y_{i}^{\mathrm{SAM}}\right)\right),$$

where $p_{i}$ denotes the predicted probability that the *i*-th sequence–annotation pair is a true match, $y_{i}^{\mathrm{SAM}}\in \left\{0,1\right\}$ is the binary indicator for the *i*-th pair (with $y_{i}^{\mathrm{SAM}}=1$ for positive pairs and 0 otherwise), α is a class balancing parameter, and γ is the focusing parameter.

#### 4.4.3. Overall Pre-Training Objective

The total pre-training loss is a weighted sum of the above objectives:

(5) $$\min_{\theta }\left[\beta_{\mathrm{HC}}L_{\mathrm{HC}}+\beta_{\mathrm{SAC}}L_{\mathrm{SAC}}+\beta_{\mathrm{SAM}}L_{\mathrm{SAM}}\right],$$

where θ includes all trainable parameters of the PLM, ALM, and projection heads, and $\beta_{\mathrm{HC}}$, $\beta_{\mathrm{SAC}}$, and $\beta_{\mathrm{SAM}}$ denote the loss weights for the three pretraining objectives.

### 4.5. Training and Downstream Evaluation Protocol

Given the significant imbalance in PPI types within the datasets, micro-F1 is preferred over macro-F1 as an evaluation metric for multi-label PPI type prediction. We divide the PPIs into training (80%) and validation (20%) sets across all baselines, as reported in previous studies [16,29]. Downstream PPI prediction experiments are repeated over five independent runs. Results are reported as the mean ± standard deviation unless otherwise stated. Model selection is performed on the validation set, and the selected configuration is evaluated on the corresponding test split. Additional architecture and optimization hyperparameters are provided in the Supplementary Materials Figures S1–S7 and Tables S1–S5.

### 4.6. Baseline Models and Comparative Settings

We compare our model against several established protein representation learning methods, including GNN-PPI [16]. In addition, we consider language models pretrained on large-scale sequence datasets, such as ESM-2 [11]. For cross-species prediction, we also include INTREPPID [43], a model designed for cross-species PPI prediction. For the HPI benchmark, we compare HIPPO with ESM-2, GNN-PPI, ProteinBERT, and INTREPPID under a unified downstream prediction setting. This revised comparison uses the same baseline group as the main PPI benchmark analyses where applicable, thereby improving consistency across tasks. For all encoder-based comparisons, the protein encoder is kept fixed, and the dataset-specific downstream PPI prediction module is trained according to the corresponding benchmark protocol.

## 5. Conclusions

In summary, HIPPO incorporates hierarchical biological relationships into protein representation learning for PPI prediction. Results from intra-species, cross-species, and HPI evaluation settings support the utility of combining protein sequences with structured biological attributes. Ablation analyses further indicate that hierarchical information contributes to predictive performance. Attention-based residue attribution provides preliminary model-interpretation evidence for interface-related residues. Overall, this study suggests that structured biological knowledge can improve representation learning for PPI prediction while highlighting the need for improved modeling of context-specific interactions.

## Figures and Tables

**Table 1:** Performance under the standard HPI split. Results are reported as the mean ± standard deviation over five independent runs. The best result in each column is shown in bold.

Encoder	Parameters	AUROC	Macro-F1	AUPRC
HIPPO (ours)	20 M	0.731±0.004	0.601±0.020	0.332±0.014
ESM-2	650 M	0.706±0.015	0.640±0.007	0.377±0.008
ProteinBERT	∼1 B	0.641±0.032	0.547±0.007	0.275±0.027
GNN-PPI	end-to-end	0.594±0.029	0.455±0.000	0.215±0.036
INTREPPID	end-to-end	0.503±0.061	0.495±0.030	0.177±0.026

**Table 2:** Family-wise AUROC under the LOVO protocol. Results are reported as the mean ± standard deviation over five independent runs. Column names abbreviate Coronaviridae, Papillomaviridae, Retroviridae, Herpesviridae, and Orthomyxoviridae. The best result for each viral family is shown in bold.

Encoder	Corona	Papilloma	Retro	Herpes	Orthomyxo
HIPPO (ours)	0.364±0.008	0.603±0.004	0.612±0.019	0.496±0.005	0.492±0.004
ESM-2	0.521±0.019	0.536±0.011	0.523±0.016	0.591±0.008	0.582±0.030
ProteinBERT	0.544±0.021	0.594±0.007	0.604±0.017	0.457±0.015	0.664±0.032
GNN-PPI	0.532±0.046	0.520±0.026	0.500±0.022	0.504±0.020	0.554±0.030
INTREPPID	0.477±0.034	0.504±0.025	0.468±0.033	0.547±0.024	0.599±0.012

**Table 3:** Number of interaction pairs in the standard HPI split. A positive-to-negative ratio of 1:5 is maintained in each split.

Split	Positive Pairs	Negative Pairs	Total Pairs
Training	6399	31,995	38,394
Validation	91	455	546
Test	625	3125	3750
Total	7115	35,575	42,690

## Data Availability

The public datasets used in this study are described in Section 4.1 and are available from UniProtKB/Swiss-Prot, Pfam, STRING, and the host–pathogen interaction datasets cited therein. The processed datasets and source code generated during the current study are available from the corresponding author upon reasonable request.

## Supplementary Materials

The following supporting information can be downloaded at: https://www.mdpi.com/article/10.3390/ijms27146242/s1.
