# Supplementary material for: Hierarchical Contrastive Learning for Protein–Protein Interaction Prediction Across Organisms
Source: Int J Mol Sci. 2026 Jul 13;27(14):6242. doi: 10.3390/ijms27146242 (PMC13410131; doi:10.3390/ijms27146242)
Supplement: Supplementary file 1 [file ijms-27-06242-s001.zip › ijms-4398754-supplementary-revised.pdf]

Supplementary Information

Hierarchical Contrastive Learning for Protein-Protein Interaction Prediction Across Organisms

Shiyi Liu, Buwen Liang,Yuetong Fang,Zixuan Jiang, Renjing Xu

Correspondence: renjingxu@hkust-gz.edu.cn

Contents

Supplementary Figure S1. Model architectures and training curves 2

Supplementary Figure S2. Performance across various data paradigms 3

Supplementary Figure S3. Model performance on various PPI types 4

Supplementary Figure S4. Feature visualization of protein representations 5

Supplementary Figure S5. Attention-based residue attribution on representative complexes 6

Supplementary Figure S6. Attention-based visualization of motif-related residues 7

Supplementary Figure S7. Attention-based visualization of residue and functional sites. 8

Supplementary Table S1. Performance comparison under ablation settings on SHS27k and SHS148k datasets 9

Supplementary Table S2. PPI prediction performance on

SHS27k and SHS148k (DFS/BFS, by difficulty) 9

Supplementary Table S3. Comparison on different PPI categories 10

Supplementary Table S4. Performance comparison on cross-species PPI prediction 10

Supplementary Table S5. Main hyperparameters used for HIPPO pretraining and downstream PPI prediction 11

Supplementary Method S1. Dataset Processing and Benchmark Splits 11

Supplementary Method S2. Attention-based Residue Attribution Analysis 12

Supplementary Method S3. Attention analysis 13

Supplementary Figure S1. Model architectures and training curves

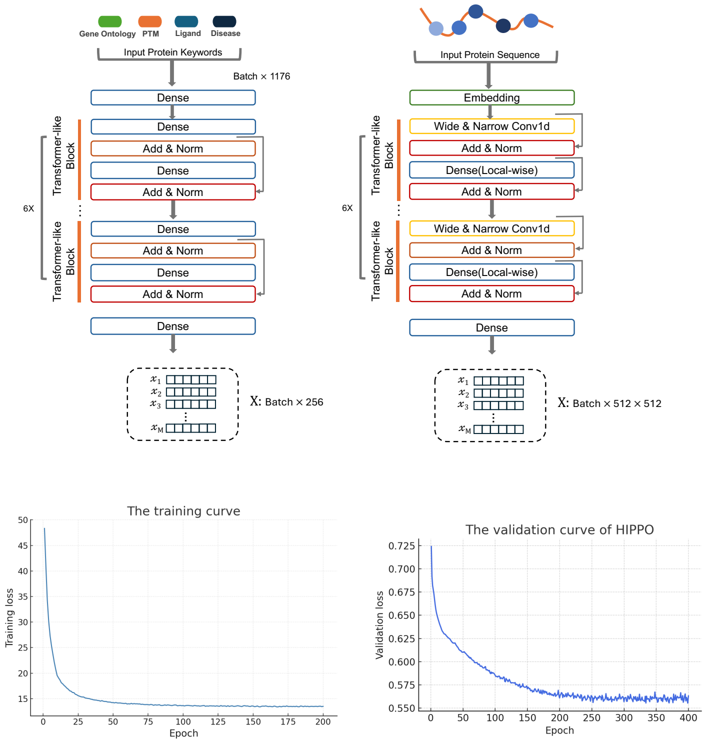

Supplementary Fig. S1. Schematic overview of the protein sequence and annotation encoding architecture within HIPPO. The input protein sequence is first embedded and then processed by six transformer-like blocks, each consisting of wide and narrow 1D convolution layers, local-wise dense (fully connected) layers, and layer normalization. The output sequence representations are aggregated by an attention pooling module to obtain a global protein embedding, which is then projected to a 256-dimensional space by a linear transformation. Both representations are further combined for downstream protein–protein interaction prediction tasks.

Supplementary Figure S2. Ablation and comparison of hierarchical and annotation components on SHS148k.

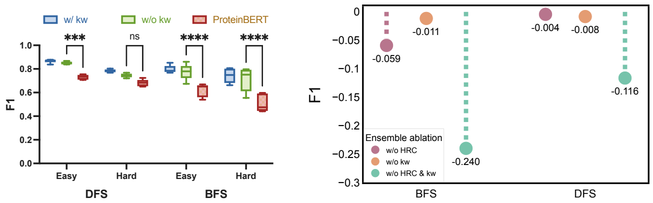

Supplementary Fig. S2. (Left) Performance comparison of our model with and without keyword annotations, as well as the baseline ProteinBERT, on the SHS148k dataset. Statistical significance between groups is indicated: **** ($p < 0.0001$), *** ($p < 0.001$), ** ($p < 0.01$). **(Right)** Relative drop in model performance (ΔF_1) on the SHS148k dataset when excluding hierarchical attributes (HRC) and/or keyword annotations (kw), as measured by F_1 score. Each point is computed from the mean F_1 across repeated runs.

Supplementary Figure S3. Performance comparison across PPI types on SHS27k

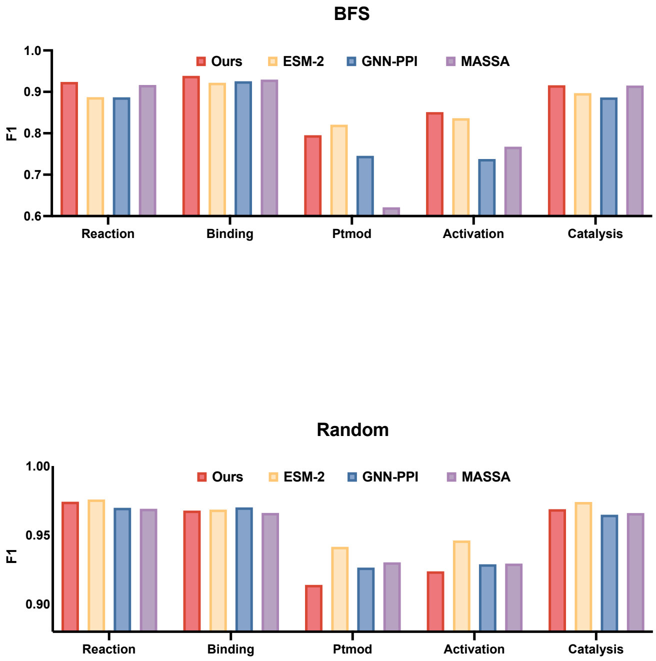

Supplementary Fig. S3. Performance comparison across PPI types on SHS27k. Bar plots showing the F1 scores of four models (Ours, ESM-2, GNN-PPI, and MASSA) across five distinct protein–protein interaction types (Reaction, Binding, PTM, Activation, and Catalysis) under (upper) BFS and (lower) random sampling strategies on the SHS27k dataset.

Supplementary Figure S4. Feature visualization

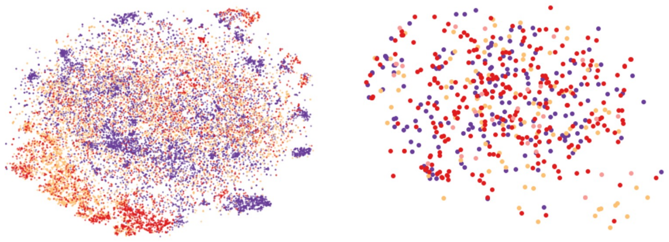

Supplementary Fig. S4. Feature visualization of protein representations. (Left) Visualization of four different domain embeddings (Protein kinase domain, WD40 repeats, Immunoglobulin domain, Ankyrin repeat). **(Right)** Visualization of three different lineage embeddings (Actinobacteria, Bacteroidetes, Firmicutes) on the Beta-lactamase family PF00144 from Pfam. All embeddings were generated using the encoder trained *without* keyword pre-training on Swiss-Prot. t-SNE projections visualize the information captured by the protein representation model.

Supplementary Figure S5. Attention-based residue attribution on representative complexes

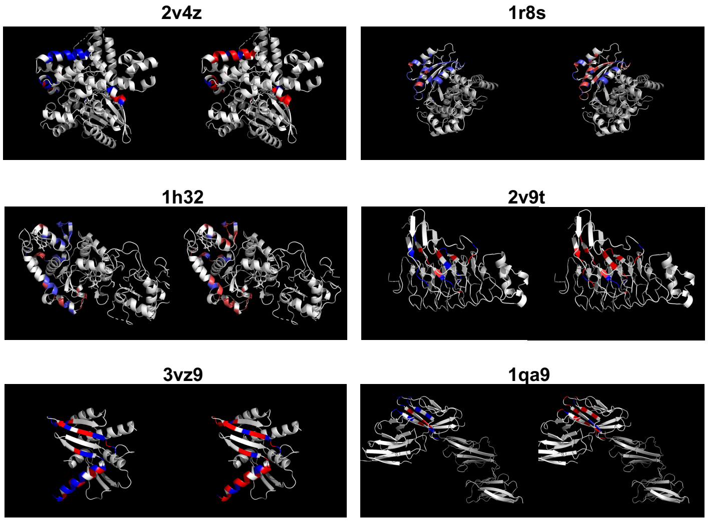

Supplementary Fig. S5. Attention-based residue attribution on representative complexes. Visualization of attention-highlighted residues on six representative protein structures (PDB IDs: 2v4z, 1r8s, 1h32, 2v9t, 3vz9, and 1qa9). For each structure, the left panel displays residues highlighted by the model trained *without* hierarchical relationship constraint (HRC) pretraining, and the right panel shows residues highlighted by the model trained *with* HRC pretraining. Red indicates attention-highlighted residues overlapping with annotated interface-related residues, whereas blue indicates highlighted residues outside the annotated set. These visualizations summarize model-attribution patterns based on overlap with annotated interface-related residues.

Supplementary Figure S6. Attention-based visualization of motif-related residues

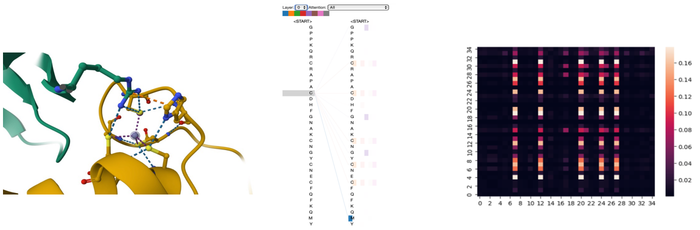

Supplementary Fig. S6. Attention-based visualization of motif-related residues. Visualization of attention weights for each amino acid residue in the Cys4 zinc-finger protein motif (PDB: 3VUX). **Left:** Three-dimensional structure of the protein, with the four zinc-coordinating residues (Cys7, Cys12, Cys24, and Cys27) highlighted. **Center:** Sequence-level attention map, showing the distribution of attention weights between individual residues. **Right:** Heatmap representation of the attention matrix, illustrating attention signals among zinc-coordinating cysteine residues.

Supplementary Figure S7. Attention-based visualization of residue and functional sites

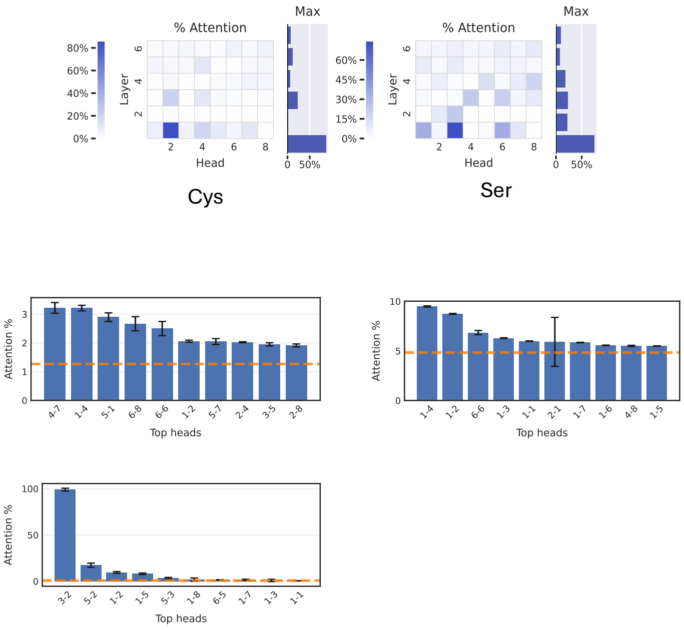

Attention head specialization for Cys and Ser residues and interpretation across functional contexts. (Top) Heatmaps depicting the proportion of attention for each model head (organized by layer and head index) that is allocated to cysteine (Cys, left) and serine (Ser, right) residues, averaged across the test set. Bar plots to the right show the maximal attention percentages observed for each head. **(Bottom row)** Bar plots displaying the percentage of attention captured by the top 10 heads (denoted as <layer>-<head>) with respect to three biological contexts: contact maps (left), binding sites (middle), and PTM (post-translational modification) positions (right), with 95% confidence intervals. Dashed orange lines indicate the expected baseline if attention were uniformly distributed across heads. These analyses summarize attention enrichment patterns associated with selected residue types and annotated functional sites.

**Dataset**	SHS27k			SHS148k		
	DFS	BFS	Random	DFS	BFS	Random
**Full**	0.8227	0.8328	0.9115	0.8673	0.8541	0.9376
**w/o HRC**	0.7753	0.7710	0.9083	0.8630	0.7951	0.9487
**w/o kw**	0.8110	0.8010	0.9030	0.8591	0.8426	0.9316
**w/o HRC & kw**	0.6231	0.6231	0.8441	0.7509	0.6140	0.9080

Supplementary Table S1. Performance comparison on the SHS27k and SHS148k datasets under various ablation settings. The table reports the results for three sampling strategies (DFS, BFS, Random) for each dataset. "Full" refers to the complete model; "w/o HRC" and "w/o kw" denote the model without hierarchical attributes and keyword annotations, respectively; "w/o HRC & kw" indicates both modules are removed. Higher values indicate better performance.

**SHS27k**	**DFS**			**BFS**		
	All	Medium/ES	Hard/NS	All	Medium/ES	Hard/NS
Ours	0.831 ± 0.003	0.843 ± 0.003	0.730 ± 0.004	0.816 ± 0.002	0.832 ± 0.002	0.708 ± 0.005
GNN-PPI	0.711 ± 0.002	0.722 ± 0.002	0.619 ± 0.003	0.803 ± 0.002	0.824 ± 0.003	0.688 ± 0.007
ESM-2	0.723 ± 0.005	0.737 ± 0.004	0.611 ± 0.007	0.806 ± 0.003	0.823 ± 0.003	0.686 ± 0.005
INTREPPID	0.736 ± 0.006	0.751 ± 0.006	0.593 ± 0.014	0.722 ± 0.022	0.742 ± 0.021	0.581 ± 0.031

**SHS148k**	**DFS**		**BFS**	
	Medium/ES	Hard/NS	Medium/ES	Hard/NS
Ours	0.866 ± 0.006	0.784 ± 0.006	0.797 ± 0.013	0.742 ± 0.025
GNN-PPI	0.852 ± 0.020	0.743 ± 0.0065	0.844 ± 0.0027	0.764 ± 0.008
ESM	0.873 ± 0.0008	0.763 ± 0.00037	0.811 ± 0.0139	0.770 ± 0.0153
INTREPPID	0.833 ± 0.0005	0.728 ± 0.0129	0.717 ± 0.016	0.555 ± 0.015

Supplementary Table S2. Detailed performance comparison on SHS27k (upper) and SHS148k (lower) datasets across all, medium/easy (ES), and hard/novel (NS) subsets under DFS and BFS sampling. The values are reported as mean ± standard deviation over repeated runs. "Ours" refers to the proposed model; results for baseline methods (GNN-PPI, ESM, INTREPPID) are also shown.

**Method**	**Reaction**	**Binding**	**Ptmod**	**Activation**	**Inhibition**	**Catalysis**	**Expression**
Ours	0.8606	0.8811	0.8899	0.7737	0.6304	0.9131	0.2209
ESM-2	0.6514	0.7957	0.6905	0.6925	0.7673	0.8412	0.3780
GNN-PPI	0.7676	0.8142	0.6616	0.7165	0.7779	0.8551	0.3515
MASSA	0.7134	0.8085	0.6851	0.7461	0.7338	0.8667	0.3771

Method	Reaction	Binding	Ptmod	Activation	Inhibition	Catalysis	Expression
Ours	0.9239	0.9387	0.7954	0.8510	0.7513	0.9159	0.5054
ESM-2	0.8871	0.9219	0.8208	0.8365	0.7308	0.8969	0.4424
GNN-PPI	0.8868	0.9258	0.7455	0.7377	0.7661	0.8864	0.4884
MASSA	0.9164	0.9292	0.6209	0.7673	0.7355	0.9149	0.4217

Supplementary Table S3. F1-score comparison of four models (Ours, ESM-2, GNN-PPI, and MASSA) across seven functional categories: Reaction, Binding, PTmod, Activation, Inhibition, Catalysis, and Expression. Higher values represent better predictive accuracy on the corresponding protein function.

Species	E. Coli	Yeast	C. elegans	Arabidopsis	Mouse	D. melanogaster
	73224	769028	3123146	4251642	4850272	1495180

Method	E. Coli	Yeast	C. elegans	Arabidopsis	Mouse	D. melanogaster
Ours	0.7559	0.7452	0.4940	0.7028	0.7177	0.5108
GNN-PPI	0.2671	0.1968	0.3831	0.3511	0.3942	0.4709
Proteinbert	0.2899	0.2182	0.2626	0.3638	0.4776	0.0600
ESM-2	0.4067	0.5079	0.4916	0.2371	0.2851	0.2598
intreppid	0.4586	0.4264	0.3533	0.2951	0.5079	0.1423
pipr	0.3898	0.4337	0.4833	0.4509	0.4408	0.4374

Supplementary Table S4. Performance comparison of six models (Ours, GNN-PPI, ProteinBERT, ESM-2, Intreppid, and pipr) on cross-species PPI prediction. The upper table lists the number of protein pairs in each species, and the lower table reports the F1 scores for each species. "Ours" denotes the proposed hierarchical model. Higher values indicate stronger generalization ability across species.

Supplementary Method S1. Dataset Processing and Benchmark Splits

This section provides additional details for the cross-species benchmark splits used to evaluate transfer across organisms. Additional dataset construction details for pretraining, intra-species PPI prediction, and host–pathogen interaction prediction are provided in the main Methods section.

To evaluate the generalization ability of our method across species, we conducted cross-species protein–protein interaction (PPI) prediction experiments. Specifically, the model was trained on the SHS148k dataset, which contains human PPI pairs, and evaluated on PPI datasets from six different species: *Escherichia coli*, yeast, *Caenorhabditis elegans*, *Arabidopsis thaliana*, mouse, and *Drosophila melanogaster*. The number of test PPIs for each species is reported in Supplementary Table S4 (top), ranging from 73,224 (E. coli) to 4,850,272 (mouse).

In each cross-species setting, the test set comprises all available PPI pairs within the corresponding species, ensuring that the model never observes these interactions during training. Performance was assessed by F1 score, and results for all compared methods are listed in Supplementary Table S4 (bottom).

The results show that HIPPO achieves the highest F1 score among the compared methods on five of the six evaluated species and ranks near the top on *C. elegans*. The highest F1 score is observed on mouse (0.7177), whereas the lowest HIPPO F1 score is observed on *C. elegans* (0.4940). These results highlight the difficulty of cross-species PPI transfer and suggest that hierarchy-aware protein representations can support transfer across several

evaluated species.

Supplementary Method S2. Attention-based Residue Attribution Analysis

For attention-based residue attribution, we used annotated protein-protein binding-site labels from the EDLMPPI dataset described by Hou *et al.* (2023). Our analysis follows the attention-based methodology proposed by Vig *et al.* (2020), which interprets self-attention patterns within protein language models and summarizes residues that receive high model-derived attention scores.

Given an input sequence $\mathcal{S} \in \mathbb{R}^{L \times d}$, the model outputs a set of self-attention matrices $A_{l,h} \in \mathbb{R}^{L \times L}$ for each encoder layer l and attention head h. To obtain residue-level attribution scores, we aggregate attention scores over empirically selected layers and heads. For each residue i, the aggregated attention score is computed as:

$$a_i = \frac{1}{N_h N_l} \sum_{h=1}^{N_h} \sum_{l=1}^{N_l} \sum_{j=1}^{L} A_{l,h}[i, j] \tag{S1}$$

where N_h and N_l represent the numbers of selected attention heads and layers, and $A_{l,h}[i, j]$ denotes the attention value from residue i to j. The top-k residues with the highest a_i are designated as attention-highlighted residues. A consensus voting scheme is applied across selected layers and heads, and residues most frequently appearing among the top-k positions are retained for overlap analysis with annotated interface-related residues.

Agreement with annotated interface-related residues is summarized using overlap score (the proportion of highlighted residues matching the annotated sites) and false positive rate (the proportion of highlighted residues not in the annotated set). As summarized in Supplementary Figure S5 and the corresponding

data table, the model trained with HRC shows higher agreement with the annotated residues than the model trained without HRC. Qualitative analysis on representative proteins (PDB IDs: 2v4z, 1r8s, 1h32, 2v9t, 3vz9, 1qa9) suggests that hierarchical pretraining can make attention-derived residue attribution more concentrated around annotated interface-related regions. These analyses provide model-attribution evidence based on overlap with annotated interface-related residues.

Supplementary Method S3. Attention analysis

To systematically interpret the attention mechanisms learned by our protein language model, we adopted and extended visualization strategies proposed in previous works by Elnaggar *et al.* (2020a). For motif-related residue analysis (**Supplementary Figure S6**), we used the `bertviz` toolkit to extract and display the attention weights between all residue pairs within a protein sequence. This approach allows us to visualize attention among residues belonging to structural motifs, such as the zinc-coordinating Cys residues in the Cys4 zinc-finger protein motif (PDB: 3VUX). Visualization of the attention matrix shows attention signals among zinc-coordinating residues (e.g., Cys7, Cys12, Cys24, and Cys27), suggesting that these residues contribute to the model's internal representation.

For residue and functional site-specific attention (**Supplementary Figure S7**), we adopted the quantitative analytical framework introduced by Vig *et al.* (2021). For each attention head in the model, we computed the percentage of total attention allocated to specific amino acid types (e.g., cysteine, serine) across the test set, as well as the proportion of attention assigned to functionally annotated positions (interface-related residues, PTMs, contact maps). For each head, the attention values were summed for the target residue type or functional label, and normalized by the total attention output of the head. By comparing these percentages with the background frequencies of the respective residues or functional sites, we summarized heads with enriched attention toward particular amino acids or annotated positions.

Together, these analyses provide mechanistic insight into how specific layers and heads within the model allocate attention to particular amino acids or annotated regions, and support a cautious interpretation of attention as a residue-attribution signal.

Supplementary Table S5. Main hyperparameters used for HIPPO pretraining and downstream PPI prediction

Setting	Value
**Protein-view pretraining**	
Maximum sequence length	512 residues
Number of amino-acid tokens	27
Number of annotation labels	1178
Hidden dimension	512
Global representation dimension	256
Encoder depth	6 layers
Attention heads	8
Attention head dimension	64
Global tokens	2
Narrow convolution kernel	9
Wide convolution kernel and dilation	kernel size 9, dilation 5
Batch size	128
Maximum pretraining epochs	100
Optimizer	AdamW
Initial learning rate	3×10^{-4}
Minimum learning rate	1×10^{-6}
Warmup learning rate and steps	1×10^{-6} for 3000 steps
Weight decay	0.05
Queue size	65,536
Sequence-annotation temperature	initialized to 0.07 and clipped to $[0.001, 0.5]$ during training
Sequence-annotation soft-target coefficient	0.4
Loss weights	1.0 for sequence-annotation contrastive, sequence-annotation matching, sequence language modeling, and annotation language modeling objectives
**Downstream PPI prediction**	
Repeated-run setting	Five independent runs
Batch size	64
Maximum epochs	400
Optimizer	Adam
Learning rate	1×10^{-6}
Weight decay	5×10^{-4}
Learning-rate scheduler	ReduceLROnPlateau, factor 0.5, patience 20
Loss function	Binary cross-entropy with logits
Prediction threshold	Sigmoid probability > 0.5
Dropout	enabled in the downstream predictor
Network-view predictor	GIN-based predictor with 2 graph convolution layers and pooling size 3
Model selection	Best validation F1 score
